# Array-CGH characterization and genotype-phenotype analysis in a patient with a ring chromosome 6

**DOI:** 10.1186/1755-8794-6-3

**Published:** 2013-02-11

**Authors:** Laura Ciocca, Cecilia Surace, Maria Cristina Digilio, Maria Cristina Roberti, Pietro Sirleto, Antonietta Lombardo, Serena Russo, Valerio Brizi, Simona Grotta, Claudio Cini, Adriano Angioni

**Affiliations:** 1 Cytogenetics and Molecular Genetics Unit, Children’s Hospital “Bambino Gesù”, IRCCS, Rome, Italy; 2 Medical Genetics Unit, Children’s Hospital “Bambino Gesù”, IRCCS, Rome, Italy; 3 Neurorehabilitation Unit, Children’s Hospital “Bambino Gesù”, IRCCS, Rome, Italy

**Keywords:** Array-CGH, Heart defects, Ring chromosome 6

## Abstract

**Background:**

Ring chromosome 6 is a rare constitutional abnormality that generally occurs *de novo*. The related phenotype may be highly variable ranging from an almost normal phenotype to severe malformations and mental retardation. These features are mainly present when genetic material at the end of the chromosome is lost. The severity of the phenotype seems to be related to the size of the deletion. About 25 cases have been described to date, but the vast majority reports only conventional cytogenetic investigations.

**Case presentation:**

Here we present an accurate cyto-molecular characterization of a ring chromosome 6 in a 16-months-old Caucasian girl with mild motor developmental delay, cardiac defect, and facial anomalies. The cytogenetic investigations showed a karyotype 46,XX,r(6)(p25q27) and FISH analysis revealed the absence of the signals on both arms of the chromosome 6. These results were confirmed by means of array-CGH showing terminal deletions on 6p25.3 (1.3 Mb) and 6q26.27 (6.7 Mb). Our data were compared to current literature.

**Conclusions:**

Our report describes the case of a patient with a ring chromosome 6 abnormality completely characterized by array CGH which provided additional information for genotype-phenotype studies.

## Background

Ring chromosome 6 [r(6)] is a rare chromosomal disorder that usually arises *de novo*[1]*.* This special shape, in most cases, is supposed to be the result of breaks and deletions at both ends of the chromosome, with a subsequent fusion of the remaining portions [2]. This event typically results in partial monosomy of the distal ends of the short and long arms of the chromosome. The formation of the ring is usually sporadic and may arise either during meiosis or in the post-zygotic period [3]. Furthermore, though uncommon, an additional mechanism has been described in literature: the telomere-telomere fusion with no detectable loss of euchromatine [4]. From the first published case of r(6) in 1973 [5], about 25 cases have been reported to date. The phenotype related to this chromosomal disorder is highly variable, and it depends on the extent of the deletions [6,7] and the stability of the ring chromosome in post-zygotic mitosis. It can range from minimal clinical signs with normal intelligence, to severe phenotypical and neurological defects [8,9]. The most frequent clinical features are growth failure, mental retardation, congenital heart defects, short neck, and typical facial anomalies including flat nasal bridge, micrognathia, and low-set ears [8]. Malformations involving the ocular, auditory and central nervous systems have also been described in affected patients [10].

Here we report a 16-months-old girl with *de novo* ring chromosome 6, identified by conventional cytogenetics. Terminal deletions on 6p and 6q were detected using BAC/PAC probes in Fluorescence In Situ Hybridization (FISH). Chromosome rearrangements were characterized by oligo array-Comparative Genomic Hybridization (array-CGH). To date, the loss of genetic material was demonstrated by means of FISH or microsatellite analysis and only in two cases the extent was exactly quantified [6,10]. We used array-CGH to establish the size of the associated deletion in the ring chromosome and to obtain more precise details for genotype-phenotype correlations.

## Case presentation

### Clinical report

The patient, a female, is the first child of healthy unrelated parents. Family history was unremarkable. The baby was born by cesarean section at the 38^th^ week of gestation. At the 34th week of pregnancy cerebral ventriculomegaly was diagnosed by fetal ultrasonography. Birth weight was 2960 g, length 48 cm, head circumference 35 cm. Apgar scores were 9 and 10 at 1 and 5 minutes, respectively. Phenotypical examination showed high nasal bridge, prominent lips, protruding tongue.

Cerebral magnetic resonance imaging (MRI) revealed enlarged lateral ventricles, particularly in correspondence to the occipital horns. Two-dimensional color-Doppler echocardiography showed an atrial septal defect ostium secundum type and a large patent ductus arteriosus. Ophthalmological examination and renal ultra-sonography were normal.

The patient underwent surgical ligation of patent ductus arteriosus at 20 days of life.

Clinical evaluation at 5 months revealed facial anomalies (prominent orbital regions, high nasal bridges, thick lips, low-set ears). Weight was 6.100 kg (3^rd^-10^th^ centile), length 62 cm (10^th^-25^th^ centile), head circumference 43 cm (75^th^-90^th^ centile). Motor developmental milestones were mildly delayed.

Re-evaluation at 16 months showed no additional phenotypical findings. Weight was 8.800 kg (3^rd^-10^th^ centile), length 76 cm (10^th^-25^th^ centile), head circumference 48 cm (75^th^-90^th^ centile). The baby was sitting at 10 months and was not able to walk without support. Audiometric examination was normal.

## Methods

### Cytogenetic analysis

Culturing, harvesting of peripheral blood cells and chromosome banding were performed according to standard methods. GTG-banded chromosomes were studied and the karyotype was interpreted according to the International System for Human Cytogenetic Nomenclature (ISCN 2009).

#### Fluorescence In Situ Hybridization (FISH)

In order to characterize the rearrangement by means of Fluorescence *In Situ* Hybridization (FISH), we used Whole Chromosome Painting (WCP) (Metasystems, Altlussheim, Germany) probe of the chromosome 6, bacterial artificial chromosome (BAC) clone specific for the region 6pter (RP11-139 J12) (start 129 360 bp – end 306 686 bp) and P1 artificial chromosome (PAC) clone for 6qter (RP1-191 N21) (start 170 844 310 bp – end 170 958 029 bp). The BAC/PAC were selected according to the UCSC Human Genome Browser (University of California Santa Cruz, February 2009 release, hg19) [11]. They were directly labeled with Cy3-dUTP (Perkin-Elmer Lifesciences, Boston, MA, USA). FISH experiments were carried out as previously described [12]. Digital images were obtained using a Nikon Eclipse E1000 epifluorescence microscope equipped with a cooled CCD camera Photometrics CoolSNAP FX. Pseudocoloring and merging of images were performed with Genikon software v3.6.16. The probes were kindly provided by Prof. Mariano Rocchi (Department of Biology, University of Bari, Bari, Italy).

#### Array-Comparative Genomic Hybridization (Array-CGH)

The array-Comparative Genomic Hybridization (aCGH) study was performed using the Agilent Human Genome CGH Microarray Kit (Agilent Technologies, Santa Clara, California, USA) following the Agilent Oligonucleotide Array-Based CGH protocol for Genomic DNA analysis (Version 6.2.1, February 2010). This platform is an oligonucleotide-based microarray that allows genome-wide survey and molecular profiling of genomic aberrations with a mean resolution of about 41 kb (60 K). The array was analyzed using the Agilent scanner, Feature Extraction software v.10.5 and Agilent Genomic Workbench Lite Edition v.6.5 (UCSC Human Genome Browser hg19). The chromosome aberrations were calculated by ADM2 algorithm. To evaluate if the Copy Number Variations (CNVs) detected were polymorphic or potentially correlated with the disease, bioinformatics analysis was carried out consulting the Database of Genomic Variants BioXRT [13].

## Results

**Figure 1:**
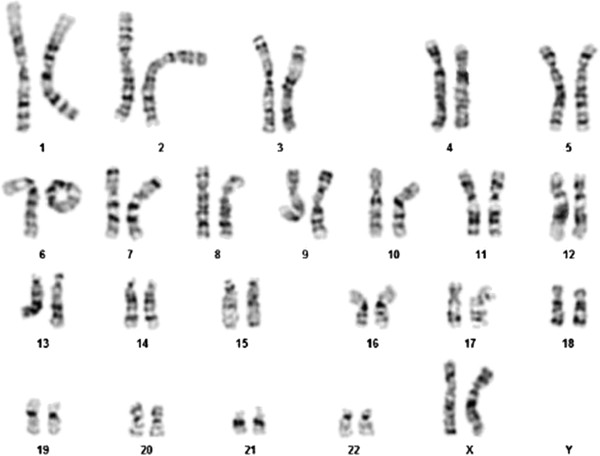
**Cytogenetic analysis.** G-banding karyotype from a peripheral blood metaphase of the patient: 46,XX,r(6).

G-banding analysis of the proband’s peripheral lymphocytes was performed at 450-bands resolution. A total of 50 cells were fully analyzed showing the following finding: 46,XX,r(6)(p25q27). The abnormal chromosome was a single monocentric ring in all the metaphases observed. No dicentric ring was found, and the size of the ring appeared to be always the same. No cells were found with two normal chromosomes 6 (Figure 1). Preliminary FISH experiment using the WCP for chromosome 6 was performed to distinguish it from other chromosomes and showed both of them entirely painted. A further FISH analysis, using the subtelomeric 6p and 6q BAC/PAC clones , displayed the absence of both signals proving that the formation of the ring results from terminal deletions involving both the long and the short arms of chromosome 6 with a following fusion of the ends of the distal segments. The positions of BAC/PAC used are more telomeric than those offered by commercial subtelomeric kits available. According to the FISH results the most plausible breakpoints could be located at the p25 and q27, proximal to the telomeric repetitive sequences.

To confirm the results obtained we performed array-CGH using Agilent platform with a mean probes distribution of about 41 kb and a high resolution in the subtelomeric regions. The analysis showed terminal deletions on 6p25.3 (1.3 Mb) and 6q26-27 (6.7 Mb), with the first conserved probe on the short arm at the position map of 1 612 710 bp start and 1 612 769 bp stop and the position of the last conserved probe on the long arm 164 112 735 bp start and 164 112 794 bp stop.

## Discussion

**Figure 2:**
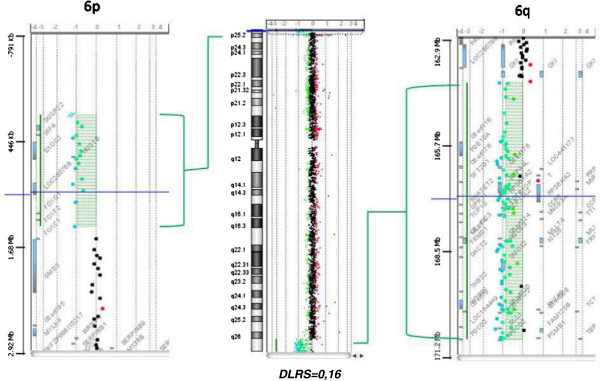
**Array-CGH results of chromosome 6.** Full view of the chromosome 6 rearrangements is displayed in the middle. Vertical dotted lines represent log2 ratio (*DLRS=0.16*). On the left and on the right are shown the magnifications of the 6p (1.3 Mb) and of the 6q (6.7 Mb) deletions.

Ring chromosome 6 is a very rare finding, generally occurring as a *de novo* event. Clinical features are manifesting with a great inter-individual variability. Most of the affected individuals show growth delay, mental retardation, facial dysmorphisms and congenital anomalies involving central nervous system (CNS), heart, ocular and auditory systems, without an identifiable phenotype. On the other hand, there are cases with minimal or absent physical anomalies, which can be associated to slight mental retardation or normal cognitive function. The presence of major congenital abnormalities can distinguish the severe from the moderate cases [10]. Previous literature reports exhibit detailed descriptions of the phenotypic features of these patients but, unfortunately, the cytogenetic analyses remain elusive due to the lack of adequate molecular investigations. Here we provide a careful and complete characterization of a ring 6 chromosome by means of FISH and array-CGH. The breakpoints were located on 6p25 and 6q26-27 and the deletion was estimated to be 1.3 Mb on the short arm and 6.7 Mb on the long arm (Figure 2).

These regions are very large and contain a great number of genes, making an appropriate genotype-phenotype correlation difficult. Nevertheless, to better understand the role of some genes included in the deleted segments, we compared our data to the two single previously reported patients with a molecular characterization of the ring chromosome 6.

The first study reported by Hökner et al. [2008] describes a woman with short stature, normal psychomotor development and minor dysmorphisms, carrying a ring chromosome 6 with very small deletions on both arms and breakpoints at 240 kb and at 190 kb from the telomeres of 6p and 6q, respectively [6]. These regions actually do not include known genes. The classic mechanism of ring formation is the breakage of both terminal chromosome arms followed by the loss of distal fragments and fusion of proximal broken ends. The severity of the phenotype seems to be related to the size of the deletion. Conversely, not all of the ring chromosomes can be associated with a loss of functional genetic material and, therefore, easily related with a clinical phenotype. These cases led to hypothesize that the ring formation produce difficulties in the sister chromatid separation at cell division, inducing the generation of secondary, lethal, aneuploid cells: this picture is called “ring syndrome” [3,10]. Owing to the lack of known genes within the deletion, the case of Höakner et al. seems to support this hypothesis.

**Figure 3:**
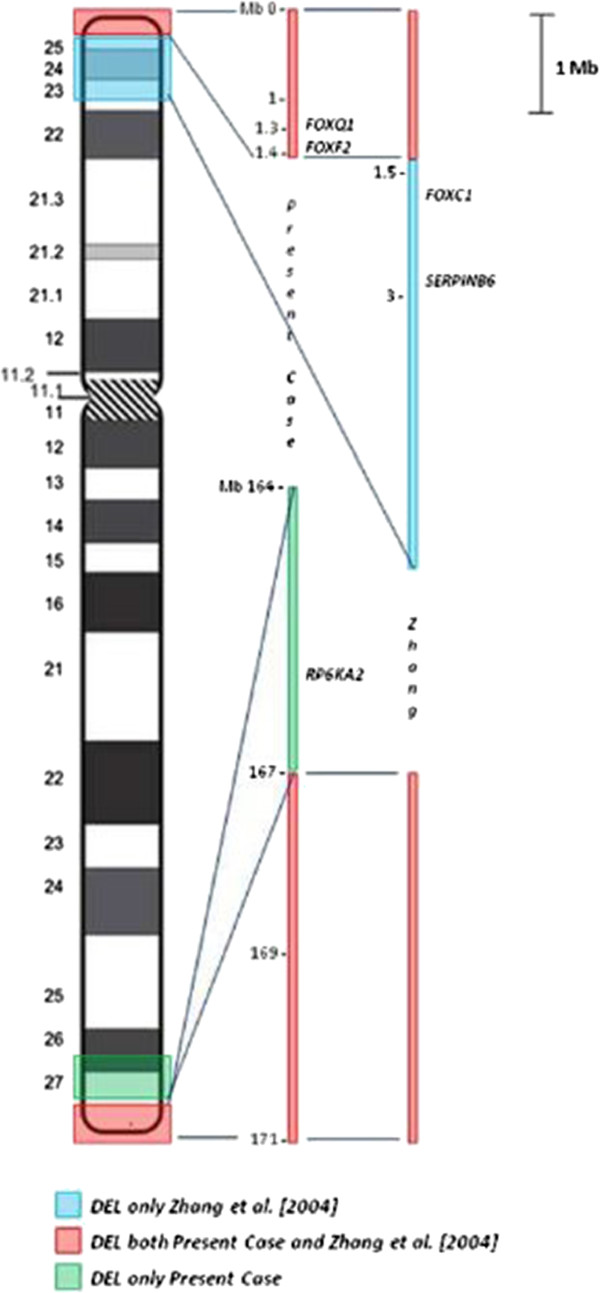
**Zoom of the deleted regions.** Schematic view of the investigated regions with evidence of the genes described.

The second study of Zhang et al. [10] is very close to our case because the deleted regions are exactly reversed and comparable in length (Figure 3). After FISH analyses the authors demonstrated a segmental deletion of about 6 Mb on 6p and 1-2 Mb on 6q [10]. Clinical features of both patients are summarized in Table 1.

**Table 1:** **Clinical features of the present patient in comparison to those of the patient reported by Zhang *et al. ***[ [10]]

	***Present case***	***Zhang et al. *[**[10]**]**
***Psychomotor delay***	+	+
***Microcephaly***	-	+
***Hydrocephalus***	-	+
***CNS malformation***	-	+
***Epilepsy***	-	+
***Cerebral ventriculomegaly***	+	+
***Eye abnormalities***	-	+
***Congenital heart defect***	+	-
***Hypotonia***	-	+
***Short/webbed neck***	-	+
***Prominent forehead***	+	+
***Flat/broad nasal bridge***	-	+
***Prominent lips***	+	-
***Protruding tongue***	+	-
***Malformed/low-set ears***	+	+
***Hearing loss***	-	+
***Wide spaced nipples***	-	+
***Growth failure***	-	+

Zhang et al. [10] provided evidences that the haploinsufficiency of the transcription factor *FOXC1*, which maps at chromosome 6p25, is essential for the development of anterior chamber anomalies in the eye in different patients [14-17]. In fact, mutations in this gene result in a range of anomalies associated with congenital glaucoma, including Axenfeld anomaly, Rieger anomaly, iris hypoplasia and Peters anomaly [10,18]. Altogether, the haploinsufficiency of the *FOXC1* gene, the consequent clinical effects observed in some cases, and the fact that this gene is not deleted in our patient, who does not show any eye anomalies, reinforce the hypothesis that *FOXC1* could be the major cause of anterior chamber eye anomalies. Furthermore, Zhang et al. proposed that the loss of the forkhead gene cluster at 6p25 (*FOXC1*, *FOXF2* and *FOXQ1*) could be responsible for CNS malformations. On the contrary, our patient displayed two of these genes deleted (*FOXF2* and *FOXQ1*), and did not reveal the same alterations. This suggests that the genes related with CNS development could be located on the long, rather than the short arm of the chromosome 6.

Another clinical sign discussed in their report is the hearing loss, a symptom that is lacking in our patient. *SERPINB6*, on 6p25, is expressed in the inner ear, particularly in hair cells. The protein encoded is a member of the serpin (serine proteinase inhibitor) superfamily and ovalbumin(ov)-serpin subfamily and plays an important role in the ear in the protection against leakage of lysosomal content during stress. A recent study, published in 2010, shows that a nonsense mutation (p.E245X) in *SERPINB6* leads to a downregulation of the gene [19]. The authors demonstrated that this mutation, introducing a premature stop-codon, destabilizes mRNA inducing its decay and reduces mRNA expression. *SERPINB6*, being located within the deleted region in Zhang’s patient, and conserved in our patient, may be a significant candidate for hearing loss related to the ring chromosome 6 disease.

The two-dimensional color-Doppler echocardiography showed that our patient had atrial septal defect ostium secondum type and a large patent ductus arteriosus, which needed surgical intervention at 20 days of life. From the investigations of the 6q deleted region we have found only one gene with a possible relationship with heart defects. *RPS6KA2* (*RSK3*) belongs to a family of four highly homologous proteins encoded by distinct genes related with Coffin-Lowry syndrome. RSKs are serine/threonine kinases, acting at the distal end of the Ras-Mitogen-Activated Protein Kinase (MAPK) signaling pathway that plays an important role in cellular events such as growth and differentiation. In humans *RSK3* is highly expressed in skeletal muscle, heart and pancreas, while, during mouse embryogenesis, its expression is increased to very high levels in the neural tube, dorsal root ganglia, the developing eye and the heart. Although no disorder associated with *RSK3* mutations is actually known, these results suggest that this gene may have a specific function in these organs [20].

Finally, our present patient shows some peculiarities in comparison with previously reported cases lacking of a detailed molecular characterization. In particular, auxological parameters are normal, whereas a growth delay is a frequent finding in ring chromosome syndromes. This may be related to the absence of secondary aneuploid cells that should lead to major ring stability. Both microcephaly and macrocephaly have been reported in ring chromosome 6, and cerebral ventriculomegaly is often associated with large head circumference as our case [21].

## Conclusions

Our report describes the most accurate and complete characterization, among the reported cases, of a ring chromosome 6. Moreover, it provides additional information not only to study genotype-phenotype but also to validate the role of already reported candidate genes and to suggest novel ones which could improve our understanding of the clinical features associated with the ring chromosome 6.

## Consent

Written informed consent was obtained from the patient’s relatives for publication of this Case report and any accompanying images. A copy of the written consent is available for review by the Editor of this journal.

## Abbreviations

r(6): Ring chromosome 6; FISH: Fluorescence *In Situ* Hybridization; array-CGH: Array-Comparative Genomic Hybridization; MRI: Magnetic Resonance Imaging; ISCN: International System for Human Cytogenetic Nomenclature; WCP: Whole Chromosome Painting; BAC: Bacterial Artificial Chromosome; PAC: P1 Artificial Chromosome; UCSC: University of California Santa Cruz, Human Genome Browser; CNVs: Copy Number Variations.

## Competing interest

The authors declare no conflict of interest.

## Authors’ contributions

LC carried out the cytogenetic analysis, FISH studies, interpretation of the data and drafted the manuscript. LC, CS and PS performed the array-CGH and the consequent bio-informatics research. MCD and CC provided the clinical evaluation. MCR and SG took care of the conception and design of the study. VB made the graphics. SR gave a contribution to the cytogenetic studies. AL participated to the critical reading of the manuscript. AA had a main role in conception and design, analyses, interpretation of the data and revised critically the manuscript. All authors read and approved the final manuscript.

## Pre-publication history

The pre-publication history for this paper can be accessed here:

http://www.biomedcentral.com/1755-8794/6/3/prepub
